# Artificial intelligence applications in biofilm research: computational approaches for biofilm analysis and antimicrobial prediction

**DOI:** 10.3389/fmicb.2026.1892814

**Published:** 2026-09-08

**Authors:** Jamil Allen G. Fortaleza, Kevin Smith P. Cabuhat, Edison D. Ramos, Jowi Tsidkenu P. Cruz, Joel G. Matamis, Jose Edwardo R. Mamaat, Amelda C. Libres, Carlos S. de Leon, Jose Jurel M. Nuevo

**Affiliations:** 1 National University, Manila, Philippines; 2 Basic Education Department, La Consolacion University Philippines, Malolos, Philippines; 3 Research Fellow, Bulacan State University, Malolos, Philippines; 4 Department of Biology, College of Science, De La Salle University, Manila, Philippines; 5 School of Medical Laboratory Sciences, St. Dominic College of Asia, Bacoor, Philippines; 6 Department of Medical Technology, Far Eastern University, Manila, Philippines; 7 College of Medical Laboratory Science, Liceo de Cagayan University, Cagayan de Oro City, Philippines; 8 Department of Medical Technology, Dr. Carlos S. Lanting College, Quezon City, Philippines; 9 College of Medical Laboratory Science, Our Lady of Fatima University, Valenzuela, Philippines

**Keywords:** artificial intelligence, biofilm-associated infections, machine learning, predictive microbiology, predictive modeling

## Abstract

Biofilms are organized groups of microbes surrounded by an extracellular polymeric substance (EPS) matrix. This structure helps microbes survive, creates metabolic differences, and makes them less sensitive to antimicrobial treatments. Because biofilms can block antimicrobials and help microbes adapt, they often cause chronic and recurring infections that are hard to treat with standard methods. Most current diagnostic and antimicrobial testing methods focus on free-floating (planktonic) microbes and do not reflect the complex structure and behavior of mature biofilms. This gap often leads to ongoing treatment failures and poor predictions of treatment outcomes. Recently, artificial intelligence (AI) and computational modeling have shown promise for improving biofilm research. These tools can help with automated detection, structural analysis, computational phenotyping, and predicting how biofilms will respond to treatments. This review examines current and emerging AI-based methods in biofilm biology, focusing on computational analysis, prediction of antimicrobial responses, and AI-supported antibiofilm therapies. It also discusses challenges such as dataset differences, limited real-world testing, difficulty understanding models, and a lack of models for clinically important mixed-species biofilms. Overall, this review shows how AI could help improve the accuracy, integration, and tailoring of biofilm research and antimicrobial management.

## 1 Introduction

Biofilm-associated infections are a major clinical challenge because the microorganisms in these communities are less responsive to antimicrobial treatments and more tolerant of the immune system (Fortaleza et al., 2024; Hernández-Huerta et al., 2025). Unlike free-floating cells, microbes in biofilms are surrounded by an extracellular polymeric substance (EPS) matrix. This matrix provides structural support, limits how far antimicrobials can reach, and creates areas with varying nutrient and oxygen levels, which help various microbes survive (Cabuhat et al., 2025; Ong et al., 2025). These factors often lead to chronic and recurrent infections that are difficult to eliminate with standard treatments (Mishra et al., 2024; Scalia and Najmi, 2025; Van Roy and Kielian, 2025). In addition to genetic antimicrobial resistance (AMR), biofilms can promote phenotypic tolerance by supporting dormant and persister cells that survive antimicrobial exposure without permanent genetic changes (Macià et al., 2018; Yang et al., 2023). Still, most microbiological diagnostics and susceptibility tests focus on planktonic cells and may not capture the complexity and diversity found in mature biofilms (Li S. et al., 2025).

These challenges highlight the need for analytical methods that can study biofilms as dynamic, organized biological systems. Many factors influence how biofilms develop and persist, including extracellular matrix structure, nutrient availability, quorum sensing, metabolic changes, and responses to environmental stress (Magana et al., 2018). To better understand biofilm-associated infections, it may be helpful to use analytical frameworks that integrate structural, molecular, and phenotypic information. Artificial intelligence (AI), especially machine learning (ML) and deep learning (DL), is attracting increasing attention because it can analyze large, complex datasets and uncover patterns that traditional methods might miss (Shen et al., 2026). Recent studies have used AI to automate biofilm detection, image segmentation, and biofilm structure analysis across different imaging platforms, thereby improving consistency and efficiency (Pérez Guerrero et al., 2025; Sengupta et al., 2025). At the same time, AI models are being tested to combine imaging, genomic, transcriptomic, and phenotypic data to better understand how biofilms develop and respond to antimicrobials (Habib et al., 2025; Verma et al., 2025; Bhattacharya et al., 2026; Dutta and Ray, 2026).

Although significant progress has been made, many AI applications in biofilm research continue to address isolated analytical tasks, such as detection, structural quantification, or antimicrobial prediction, rather than integrating these elements into comprehensive analytical frameworks. Furthermore, numerous studies are constrained by simplified experimental models, limited datasets, and inadequate validation under clinically relevant conditions. The complex relationships among biofilm architecture, metabolic heterogeneity, and antimicrobial response remain insufficiently characterized. This narrative review examines recent advances in AI applications within biofilm research, emphasizing AI-assisted detection, computational analysis of biofilm structure, and predictive modeling of antimicrobial response. Additionally, the review outlines current methodological limitations, translational challenges, and prospective directions for developing more integrative computational approaches in biofilm biology.

## 2 Biofilm-associated infections: a persistent clinical challenge

### 2.1 Clinical burden of biofilm-associated infections

Biofilm-associated infections present a significant challenge in contemporary healthcare. Microbial communities adhering to biotic and abiotic surfaces demonstrate increased tolerance to antimicrobial therapies and host immune responses (Edmiston et al., 2016; Hurlow et al., 2025). These infections substantially contribute to chronic disease progression, therapeutic failure, extended hospitalization, and elevated healthcare expenditures. Biofilms are most commonly linked to chronic wounds, implanted medical devices, ventilator-associated pneumonia (VAP), and healthcare-associated infections in intensive care units. In these clinical contexts, biofilm formation facilitates persistent microbial colonization, recurrent infections, and diminished therapeutic effectiveness (Rather et al., 2021). Table 1 summarizes the principal clinical settings, predominant pathogens, biofilm characteristics, associated outcomes, and current management limitations.

**TABLE 1:** Clinical context, biofilm characteristics, and system-level limitations in ESKAPE (*Enterococcus faecium*, *Staphylococcus aureus*, *Klebsiella pneumoniae*, *Acinetobacter baumannii*, *Pseudomonas aeruginosa*, and *Enterobacter* spp.)-driven biofilm-associated infections.

Clinical context	Dominant ESKAPE pathogens	Major biofilm characteristics	Clinical consequence	System-level limitations	References
Chronic wounds	*S. aureus*, *Pseudomonas aeruginosa*	Polymicrobial biofilms with dense EPS and hypoxic microenvironments	Delayed healing, chronic inflammation, recurrent infection	Poor antibiotic penetration and lack of biofilm-specific diagnostics	Roy et al., 2020; Keim et al., 2024
Device-associated infections (catheters and implants)	*S. epidermidis*, *S. aureus*, *E. faecium*	Rapid surface adhesion and persistent mature biofilms	Implant failure, bloodstream infection, chronic colonization	Device removal often required; antibiotics have limited efficacy	Khatoon et al., 2018; Zheng et al., 2018
VAP	*P. aeruginosa*, *Acinetobacter baumannii*, *K. pneumoniae*	Endotracheal tube biofilms enriched with multidrug-resistant cells	Increased mortality, prolonged ventilation, secondary infection	Biofilm reservoirs are difficult to detect and eradicate	Yang and Guglielmo, 2007; Alves et al., 2023; AbdelHalim et al., 2024
ICU-acquired infections	*A. baumannii*, *Enterobacter* spp., *K. pneumoniae*	Persistent healthcare surface biofilms	Nosocomial outbreaks, prolonged hospitalization, increased healthcare burden	Disinfection challenges and extensive antimicrobial resistance	Ali Alshuqayfi Snr et al., 2025; Chakraborty et al., 2025; Mukhopadhyay et al., 2025

Chronic wound infections are among the most thoroughly investigated manifestations of biofilm-associated disease. Persistent biofilms produced by *Staphylococcus aureus* and *Pseudomonas aeruginosa* impede tissue repair and prolong inflammation. They also promote recurrent microbial colonization, frequently leading to extended treatment durations and suboptimal clinical outcomes (Roy et al., 2020; Keim et al., 2024; Admella et al., 2025; Shen et al., 2025). Current management strategies include wound debridement, antimicrobial therapy, and adjunctive approaches such as antimicrobial peptides (AMP) and bacteriophage-based interventions. However, complete eradication of mature biofilms remains challenging (Aswathanarayan et al., 2022; Drago et al., 2024).

Medical devices such as urinary catheters, central venous catheters, prosthetic joints, and implanted biomaterials provide surfaces conducive to microbial attachment and subsequent biofilm formation (Khatoon et al., 2018; Zheng et al., 2018; Bouhrour et al., 2024). Device-associated infections are predominantly caused by *S. aureus*, *Staphylococcus epidermidis*, *Enterococcus faecium*, and *Klebsiella pneumoniae*. These infections often result in chronic colonization, bloodstream infections, and device malfunction or failure (Khatoon et al., 2018; Zheng et al., 2018). While antimicrobial-coated materials and surface modifications have been introduced to mitigate microbial attachment, sustained prevention of biofilm formation remains a considerable challenge (Akbari and Kjellerup, 2015; Akay and Yaghmur, 2024).

Ventilator-associated pneumonia remains a leading cause of morbidity and mortality among patients receiving mechanical ventilation (Diaconu et al., 2018). Endotracheal tubes are rapidly colonized by biofilm-forming pathogens, notably *P. aeruginosa*, *Acinetobacter baumannii*, and *K. pneumoniae*. These tubes serve as reservoirs for repeated microbial dissemination into the lower respiratory tract (Yang and Guglielmo, 2007; Alves et al., 2023; AbdelHalim et al., 2024; Codru et al., 2025). Preventive interventions include antimicrobial-coated tubes, oral hygiene protocols, and subglottic secretion drainage. These measures reduce the risk of infection but have not eliminated ventilator-associated pneumonia as a significant healthcare concern (Codru et al., 2024).

Biofilms developing on medical devices and environmental surfaces facilitate the persistence and transmission of multidrug-resistant organisms. Species such as *A. baumannii*, *Enterobacter* spp., and *K. pneumoniae* can survive in adverse environmental conditions. They can persist on healthcare surfaces, thereby increasing the risk of nosocomial outbreaks and prolonged hospitalization (Taner et al., 2024; Ali Alshuqayfi Snr et al., 2025; Chakraborty et al., 2025; Mukhopadhyay et al., 2025). As a result, infection-control strategies now prioritize device stewardship, environmental decontamination, and the advancement of biofilm-resistant materials to limit microbial persistence in healthcare facilities (Maroju et al., 2026).

### 2.2 ESKAPE pathogens and biofilm persistence

Many biofilm-related infections persist primarily because of ESKAPE pathogens, a group of clinically important microorganisms that combine multidrug resistance with robust biofilm-forming capabilities (Patil et al., 2021; Sarkar et al., 2024). These pathogens can rapidly adapt to both biotic and abiotic environments, enabling prolonged survival on medical devices, healthcare surfaces, and within host tissues (Uchil et al., 2021; Josephs-Spaulding and Singh, 2021). A comparative overview of the major biofilm mechanisms, adaptive survival traits, resistance characteristics, and translational relevance of ESKAPE pathogens is presented in Table 2.

**TABLE 2:** Comparative biofilm behaviors, resistance traits, and translational characteristics of major ESKAPE pathogens.

Pathogen	Dominant biofilm mechanism	Adaptive survival strategy	Polymicrobial interactions	Resistance and biofilm traits	Unique mechanistic feature	AI and computational relevance	References
*P. aeruginosa*	Quorum sensing-driven EPS production and biofilm maturation	Metabolic flexibility under nutrient and oxygen limitation	Competitive and cooperative interactions with *S. aureus* and *Candida* spp.	Intrinsic tolerance and biofilm persistence	Quorum sensing and EPS architecture govern structural resilience	AI modeling of spatial organization, quorum sensing, and antimicrobial diffusion	Fortaleza et al., 2025; Grace et al., 2025; Vijayakumar et al., 2025
*S. aureus*	Adhesin-mediated attachment and persister-cell formation	Metabolic dormancy and immune evasion	Frequently coexists with *P. aeruginosa*	MRSA-associated resistance and vancomycin tolerance	Dispersin B promotes biofilm dispersal	AI prediction of biofilm maturation, resistance, and persister dynamics	Ong et al., 2025; Scaffo et al., 2025
*A. baumannii*	Bap-mediated adhesion and surface biofilm formation	High desiccation tolerance and abiotic persistence	Emerging polymicrobial interactions	Carbapenem resistance and healthcare persistence	Biofilms drive ICU persistence and outbreaks	AI-assisted outbreak prediction and environmental surveillance	Fortaleza et al., 2026; Uddin et al., 2026
*K. pneumoniae*	Capsule-associated biofilm and extracellular matrix production	Capsule-mediated stress protection	Mixed-species biofilm interactions	Biofilm-linked carbapenem and cephalosporin resistance	Carbapenemase production enhances persistence	AI prediction of resistance spread and treatment failure	Li and Ni, 2023; Uddin et al., 2026
*E. faecium*	Structured biofilm maturation and surface colonization	Survival under antimicrobial and environmental stress	Polymicrobial biofilm participation	VRE-associated biofilm tolerance	Protease-based dispersal shows antibiofilm potential	AI prediction of resistance emergence and combination therapies	Pandey et al., 2021; Uddin et al., 2026
*Enterobacter* spp.	Surface biofilm formation and β-lactamase activity	Persistence in nutrient-limited hospital environments	Mixed-species biofilm interactions	Biofilm-mediated antimicrobial resistance	Farnesol emulsions exhibit antibiofilm activity	AI modeling of emerging resistance and polymicrobial behavior	Jordan et al., 2026; Uddin et al., 2026

#### 2.2.1 Pseudomonas aeruginosa

*P. aeruginosa* is recognized as one of the most clinically significant biofilm-forming pathogens, frequently associated with chronic wounds, ventilator-associated pneumonia, catheter-associated infections, and chronic respiratory diseases (Yang and Guglielmo, 2007; Alves et al., 2023; Verdial et al., 2023; AbdelHalim et al., 2024; Mishra et al., 2024). Standard treatments include antipseudomonal β-lactams, fluoroquinolones, aminoglycosides, and polymyxins; however, their effectiveness is often reduced due to multidrug resistance and biofilm-associated persistence (Mishra et al., 2024). In polymicrobial infections, interactions with *S. aureus* and *Candida* spp. can further modify biofilm architecture, virulence expression, and treatment outcomes (Kumar and Ting, 2015; Magalhães et al., 2017; Sørensen et al., 2025). Recent developments in AI have facilitated predictive modeling of quorum-sensing networks, antimicrobial diffusion, and spatial biofilm organization, thereby enhancing therapeutic decision-making and optimizing antibiofilm strategies (Fortaleza et al., 2025; Grace et al., 2025; Vijayakumar et al., 2025).

#### 2.2.2 Staphylococcus aureus

*S. aureus* is a primary etiological agent of chronic wound infections, prosthetic joint infections, catheter-associated infections, and infective endocarditis (Xu et al., 2024). Methicillin-resistant *S. aureus* (MRSA) strains pose a substantial clinical challenge due to their extensive resistance to conventional antibiotics and their ability to persist within mature biofilms. Standard therapeutic options include vancomycin, daptomycin, linezolid, and newer anti-MRSA agents; however, treatment failure remains prevalent in chronic biofilm-associated infections (Xu et al., 2024). The coexistence of *S. aureus* with *P. aeruginosa* in polymicrobial biofilms can further influence antimicrobial susceptibility and virulence traits. AI-driven methodologies are increasingly utilized to predict biofilm maturation, identify resistance patterns, and model persister-cell dynamics, thereby supporting precision antimicrobial therapy (Ong et al., 2025; Scaffo et al., 2025).

#### 2.2.3 Acinetobacter baumannii

*A. baumannii* is closely linked to ICU-acquired infections, ventilator-associated pneumonia, bloodstream infections, and healthcare-associated outbreaks (Hamidian and Nigro, 2019; Javadi, 2024; Mukhopadhyay et al., 2025; Morena et al., 2026). Its capacity to withstand desiccation and persist on hospital surfaces contributes to prolonged environmental contamination and nosocomial transmission. Therapeutic options are increasingly limited due to the global rise of carbapenem-resistant strains. As a result, treatment frequently depends on polymyxins, sulbactam-containing regimens, or combination therapies. AI-based surveillance systems have shown promise for outbreak prediction, transmission tracking, and environmental risk assessment, thereby supporting infection-control initiatives in healthcare settings (Fortaleza et al., 2026; Uddin et al., 2026).

#### 2.2.4 Klebsiella pneumoniae

*K. pneumoniae* frequently causes catheter-associated urinary tract infections, ventilator-associated pneumonia, bloodstream infections, and device-related infections (Singh et al., 2019; De Oliveira Júnior and Franco, 2020; Jia and Guo, 2021; Li and Ni, 2023). The emergence of carbapenemase-producing strains has substantially complicated clinical management by diminishing the efficacy of many first-line therapies (Ashwath et al., 2022; Bereanu et al., 2024). Biofilm formation further promotes prolonged colonization and treatment failure, especially in patients with indwelling medical devices. Recent studies have explored machine learning models for predicting antimicrobial resistance patterns, treatment outcomes, and the spread of carbapenem-resistant lineages in healthcare settings (Li and Ni, 2023; Uddin et al., 2026).

#### 2.2.5 Enterococcus faecium

*E. faecium* is commonly isolated from catheter-associated infections, prosthetic-device infections, and healthcare-associated bloodstream infections (Zirakzadeh and Patel, 2006; Paganelli et al., 2015; Kathi, 2024; Sadaqa et al., 2025). The rise of vancomycin-resistant enterococci (VRE) has markedly reduced available therapeutic options, often requiring the use of linezolid, daptomycin, or combination regimens. Ongoing colonization of healthcare environments further facilitates transmission and recurrent infection. Computational methods are increasingly being investigated to predict the evolution of resistance, identify effective antimicrobial combinations, and support individualized treatment strategies (Pandey et al., 2021; Uddin et al., 2026).

#### 2.2.6 *Enterobacter* spp.

*Enterobacter* spp. are opportunistic pathogens frequently implicated in healthcare-associated infections, especially among critically ill and immunocompromised patients (Pereira et al., 2016; Ilham et al., 2023; Kathi, 2024). Their clinical relevance is primarily attributed to β-lactamase production and the ability to form persistent biofilms on medical devices and hospital surfaces. Treatment is often complicated by inducible AmpC β-lactamase expression and multidrug resistance. Emerging AI frameworks are under investigation for predicting antimicrobial susceptibility, conducting resistance surveillance, and modeling polymicrobial biofilm behavior in complex clinical settings (Jordan et al., 2026; Uddin et al., 2026).

### 2.3 Other clinically relevant non-eSKAPE biofilm-forming pathogens

While ESKAPE pathogens account for a substantial proportion of healthcare-associated biofilm infections, numerous non-ESKAPE microorganisms also contribute significantly to the overall disease burden. *Candida albicans* is a major fungal biofilm-forming pathogen, commonly associated with catheter-related bloodstream infections, prosthetic device infections, and mixed-species biofilms involving bacterial pathogens. Infections caused by biofilm-associated *Candida* frequently exhibit reduced susceptibility to antifungal agents and are a major factor in persistent healthcare-associated infections (Akbari and Kjellerup, 2015).

*Helicobacter pylori* has been shown to form biofilm-like communities within the gastric mucosa, potentially contributing to chronic colonization, treatment failure, and recurrent infection (Hathroubi et al., 2018). *Streptococcus mutans* is a primary driver of dental plaque biofilms and dental caries, which are among the most prevalent biofilm-associated diseases globally (Zayed et al., 2021). Other clinically significant biofilm-forming organisms include *Cutibacterium acnes*, frequently implicated in prosthetic joint and implanted medical device infections, and non-tuberculous mycobacteria, which can colonize healthcare water systems and medical equipment, resulting in healthcare-associated infections that are difficult to treat (Coenye et al., 2021). Collectively, these organisms illustrate the broad taxonomic diversity of biofilm-forming pathogens and underscore that biofilm-associated diseases are not limited to the traditional ESKAPE group.

## 3 Challenges in biofilm diagnosis and treatment

Traditional microbiology and infectious disease methods mostly focus on planktonic models, treating microorganisms as free-floating, uniform populations rather than as organized, diverse communities (Damyanova et al., 2024). These methods work well for many acute infections but often fail to capture the complexity of biofilm-associated diseases. This gap can lead to discrepancies between lab test results and real clinical outcomes, which may result in ongoing infections, recurrent colonization, and less effective treatments. Many standard diagnostic and susceptibility tests still rely on fixed-endpoint measurements that do not capture the dynamic, adaptive nature of biofilm systems.

A key problem is that routine culture-based tests often miss microorganisms hidden in biofilms, especially when they are in different physiological states. Viable but non-culturable (VBNC) cells pose a major challenge because they remain metabolically active yet cannot be detected by standard culture methods (Fakruddin et al., 2013; Zhao et al., 2017; Lee et al., 2026). This means the true number of microbes may be underestimated, even when harmful populations remain in infected tissues or on medical devices (Foreman et al., 2013; König et al., 2023). Dormant persister cells can also survive antibiotics by slowing their metabolism, making them less sensitive to drugs that target active cells (Pasquaroli et al., 2014). These cells can become active again when conditions improve, which may lead to infections coming back after they seem to be cured (Wagley et al., 2021). Overall, these issues show that endpoint-based tests are limited in measuring biofilm persistence and how microbes adapt over time.

Advanced imaging modalities, including confocal laser scanning microscopy (CLSM) and fluorescence *in situ* hybridization (FISH), have enhanced structural visualization of biofilms, particularly in research settings (Barbosa et al., 2023; Neu and Kuhlicke, 2026). Despite these advancements, such techniques are limited by the need for specialized equipment, invasive sampling, and restricted scalability for routine clinical use. Furthermore, traditional biofilm imaging interpretation is predominantly descriptive, offering minimal insight into metabolic activity, antimicrobial susceptibility, or therapeutic response. Recent developments in AI-assisted methodologies aim to overcome these challenges by integrating imaging-derived structural data with computational analysis and predictive modeling (Wang Y. et al., 2025). Machine learning algorithms, convolutional neural networks (CNNs), and multi-modal computational frameworks facilitate the extraction of spatial features from imaging datasets and the integration of phenotypic and molecular information into comprehensive analytical systems (Vaz and Balaji, 2021). While these technologies are still under development, they signify a transition from descriptive visualization to data-integrated interpretation of biofilm behavior.

Therapeutic decision-making remains constrained by reliance on planktonic susceptibility metrics, such as minimum inhibitory concentration (MIC) testing. These assays do not adequately account for diffusion barriers, altered metabolic states, or the protective EPS microenvironments characteristic of mature biofilms, often leading to underestimating the antimicrobial concentrations needed for effective eradication (Song et al., 2016; Abdelghafar et al., 2022). As a result, antimicrobial regimens based primarily on planktonic MIC values may fail to reliably predict treatment outcomes in biofilm-associated infections (Broussou et al., 2018). Notably, antimicrobial susceptibility within biofilms is spatially heterogeneous and influenced by factors such as nutrient gradients, oxygen availability, EPS density, and metabolic activity across distinct regions of the microbial community. These complexities are not captured by single-variable susceptibility frameworks. Accordingly, AI-assisted predictive systems are being investigated to incorporate spatial heterogeneity, temporal adaptation, and multimodal biological data into models of antimicrobial response.

Biofilm-associated infections are more complex due to the way biofilms are organized in both space and time. Rather than being static, biofilms are dynamic microbial ecosystems. Differences in nutrients, oxygen, signaling molecules, and metabolic byproducts create local changes in gene expression, quorum sensing, metabolic activity, and microbial responses to antimicrobials within the same biofilm (Bridier et al., 2017; Lou et al., 2025). These small environments are always changing due to matrix remodeling, cell-to-cell signaling, competition, and cooperation among different species (Miller et al., 2019).

Even with recent progress, there are still major challenges in applying these findings to clinical practice. Right now, there are no widely accepted or scalable tools that can accurately measure biofilm levels or predict how they will respond to treatment in different clinical settings. Many AI models face problems such as limited data diversity, insufficient testing in mixed-species systems, limited ability to operate across both lab and real-world biofilms, and difficulties with understanding and using the models in clinical settings (Xu et al., 2025). Also, important factors such as immune responses, fibrin buildup, and specific tissue environments are often missing from training data, which can limit how well these models perform in practice. New methods such as blocking quorum sensing, breaking down biofilms with enzymes, using nanotechnology for drug delivery, and AI-based predictions look promising, but they are not yet used routinely in clinical practice because they require further testing, standardization, and regulatory approval (Empitu et al., 2025). Table 3 highlights the main differences between traditional clinical methods and new AI-based approaches for diagnosing biofilms and making treatment decisions.

**TABLE 3:** Comparison between conventional clinical approaches and AI-integrated frameworks in biofilm diagnosis and antimicrobial decision-making.

Aspect	Conventional clinical approaches	AI-integrated frameworks	References
Biofilm diagnosis	Conventional microscopy, culture-based diagnostics, and standard imaging often fail to capture the structural heterogeneity, polymicrobial organization, and physiological complexity of mature biofilms. In contrast, CLSM and structured illumination microscopy (SIM) provide high-resolution, time-resolved visualization of biofilm architecture, spatial organization, and dynamic development.	Integrate machine learning algorithms, automated image analysis, and computational feature extraction to enable rapid, high-dimensional, and more accurate identification of biofilm-forming pathogens and structural biofilm characteristics.	Moskowitz et al., 2004; Mishra et al., 2024; Bhattacharya et al., 2026 Mallya et al., 2019; Haagensen et al., 2010
Turnaround time	Diagnostic workflows are labor-intensive and heavily dependent on microbial culturing, often requiring several days before therapeutic decisions can be implemented.	AI-integrated pipelines combine automated microscopy, genomic interpretation, predictive analytics, and computational modeling to substantially reduce turnaround times and enable near real-time clinical decision support.	Avershina et al., 2023; Abbara et al., 2025; Ghedia et al., 2026
Antimicrobial decision-making	Therapeutic selection is largely empirical and based on planktonic susceptibility testing such as MIC assays, which inadequately reflect biofilm-specific resistance, metabolic dormancy, and diffusion barriers.	AI-driven predictive models integrate imaging, resistance datasets, phenotypic assays, and biofilm-specific resistance patterns to optimize antimicrobial selection and predict treatment response under biofilm-associated conditions.	Bhattacharya et al., 2026
Precision and personalization	Limited capacity to tailor interventions according to spatial heterogeneity, metabolic stratification, or biofilm-specific resistance mechanisms.	AI enables precision-oriented therapeutic strategies through personalized antimicrobial prediction, adaptive treatment modeling, and targeted biofilm-disruption approaches.	Talianu et al., 2026
Spatial and ecological interpretation	Biofilms are commonly interpreted using reductionist and planktonic-centered frameworks with minimal ecological or spatial context.	AI frameworks provide spatially resolved and systems-level interpretation by integrating structural imaging, metabolic gradients, ecological interactions, and multi-modal datasets into unified predictive systems.	Mishra et al., 2024; Bhattacharya et al., 2026; Rahman et al., 2026
Data integration	Primarily dependent on isolated laboratory variables and endpoint-based measurements that inadequately capture dynamic microbial adaptation.	Multi-modal AI systems integrate imaging, transcriptomics, metabolomics, phenotypic assays, and environmental datasets for continuous and predictive interpretation of biofilm behavior.	Mishra et al., 2024; Bhattacharya et al., 2026; Rahman et al., 2026; Talianu et al., 2026
Efficiency and scalability	Conventional workflows are resource-intensive, operator-dependent, and prone to interobserver variability and workflow inconsistency.	AI-assisted frameworks streamline analytical workflows, improve reproducibility, reduce human error, and enhance scalability across large datasets and high-throughput analytical platforms.	Mishra et al., 2024; Bhattacharya et al., 2026; Rahman et al., 2026; Talianu et al., 2026
Detection of latent microbial states	Limited ability to detect VBNC cells, dormant persisters, and metabolically heterogeneous subpopulations associated with recurrent infection.	AI-assisted modeling and multi-modal analysis improve detection and inference of hidden microbial phenotypes linked to persistence, antimicrobial tolerance, and recurrence.	Mishra et al., 2024; Bhattacharya et al., 2026; Rahman et al., 2026; Talianu et al., 2026
Predictive Capability	Primarily descriptive and retrospective, with limited capacity for forecasting resistance emergence, biofilm evolution, or therapeutic outcomes	AI frameworks support predictive and dynamic modeling of antimicrobial penetration, resistance evolution, biofilm growth, and therapeutic efficacy.	Mishra et al., 2024; Bhattacharya et al., 2026; Rahman et al., 2026; Talianu et al., 2026
Major limitations	Difficulty in identifying microbial species within polymicrobial biofilms and inability to fully capture spatial, ecological, and physiological complexity.	AI implementation remains constrained by dataset heterogeneity, limited prospective validation, model interpretability challenges, and regulatory barriers to clinical implementation.	Mishra et al., 2024; Bhattacharya et al., 2026; Rahman et al., 2026; Talianu et al., 2026

## 4 Computational phenotyping of biofilm structure and dynamics

Biofilms are structurally complex microbial communities in which EPS organization, nutrient diffusion, environmental gradients, and microbial interactions collectively determine persistence and antimicrobial tolerance (Wang Y. et al., 2025). As these processes span interconnected spatial and physiological scales, analyzing imaging, omics, or phenotypic datasets in isolation often yields only partial understanding of biofilm behavior. Consequently, computational approaches are increasingly employed to integrate heterogeneous datasets into unified analytical frameworks that connect structural observations with functional microbial activity.

**FIGURE 1:**
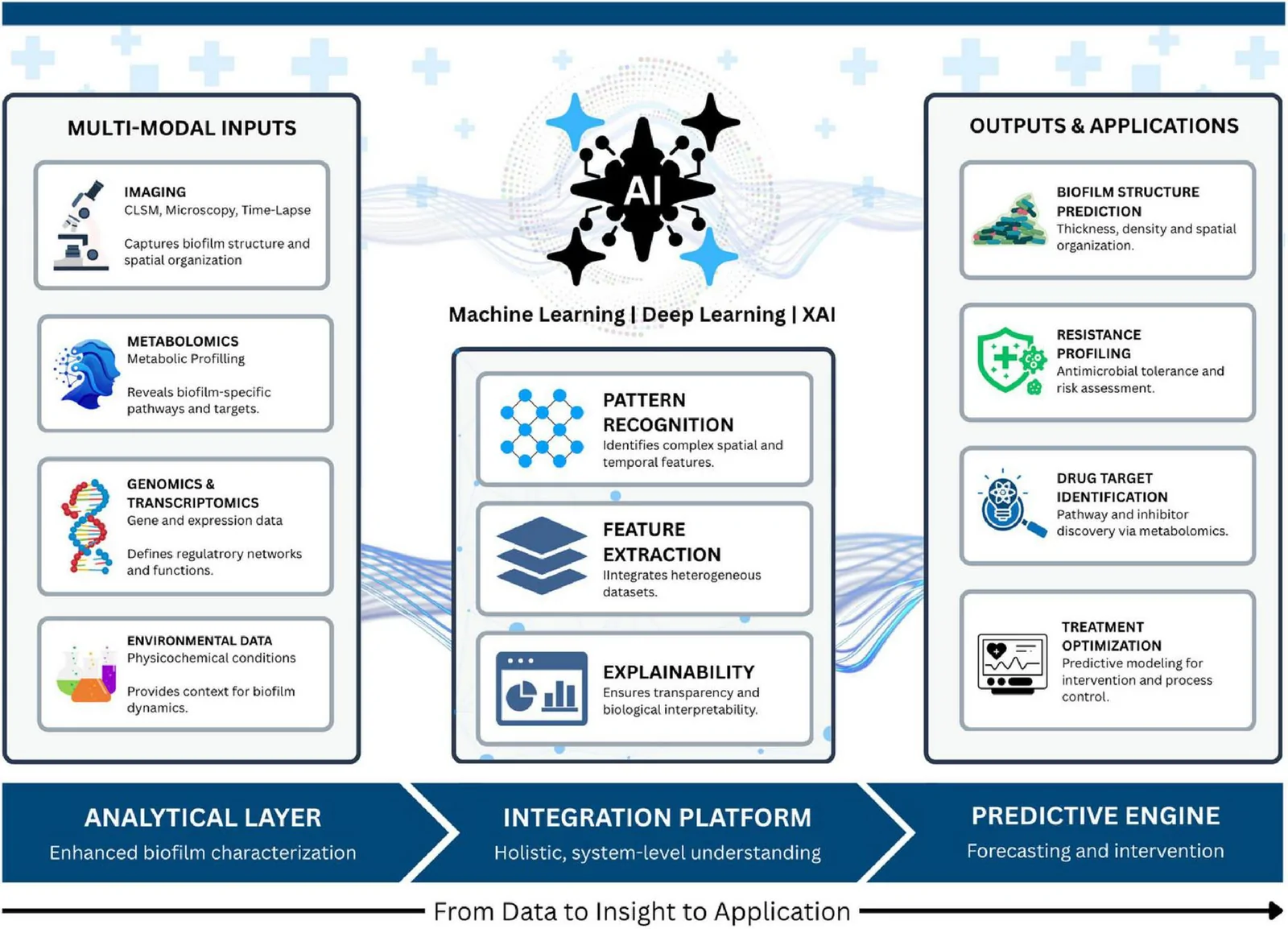
AI for biofilm analysis and precision antimicrobial response prediction.

As shown in Figure 1, computational biofilm analysis typically begins with the acquisition of multi-modal data from imaging platforms, omics technologies, metabolomic profiling, phenotypic assays, and environmental measurements. These datasets are then analyzed using ML and DL algorithms to identify biologically relevant patterns across structural, molecular, and ecological dimensions. This methodological shift has transformed biofilm characterization from primarily qualitative, microscopy-based observations to quantitative, automated computational analyses. While traditional methods such as colony-forming unit enumeration and crystal violet staining remain valuable, they are often limited by low throughput, operator dependency, and restricted ability to resolve structurally heterogeneous biofilm communities (Funari and Shen, 2022; Mishra et al., 2024).

CNNs have demonstrated strong performance in automated biofilm recognition and classification. CNN-based systems applied to rhinocytological microscopy have successfully identified chromatic and morphological features associated with biofilm formation with minimal operator intervention (Dimauro et al., 2020). Similarly, U-Net architectures trained on intraoral photographic datasets have enabled automated segmentation of dental biofilms without disclosing agents, achieving reported accuracies exceeding 91% under controlled imaging conditions (Andrade et al., 2023; Sobrinho et al., 2025). More advanced instance-segmentation frameworks, including Mask R-CNN and YOLACT, have further facilitated rapid quantification of biofilm-associated structures from scanning electron microscopy (SEM) datasets, supporting high-throughput analyses of both biotic and abiotic surfaces (Mishra et al., 2024). Collectively, these approaches improve analytical reproducibility while reducing observer-dependent variability.

Computational phenotyping has expanded the quantitative assessment of biofilm architecture. Advanced imaging modalities, including confocal laser scanning microscopy (CLSM), acoustic microscopy, and optical coherence tomography (OCT), facilitate reproducible measurement of biofilm thickness, biomass, porosity, and structural irregularity across multiple spatial scales (Good et al., 2006; Frühauf et al., 2022). Complementary image-processing techniques, such as RGB-based quantification, offer scalable and non-invasive methods for structural assessment with reduced manual variability (Asgharnejad et al., 2021). Quantitative descriptors, including roughness coefficients, fractal dimensions, and textural entropy, convert structural heterogeneity into measurable computational parameters derived from CLSM datasets (Beyenal et al., 2004). Furthermore, OCT enables visualization of subsurface architectures, such as pore networks and diffusion channels, which influence nutrient transport and antimicrobial penetration within biofilm matrices (Rosenthal et al., 2018).

Computational classification systems have improved the assessment of biofilm maturity and polymicrobial organization. Analysis of atomic force microscopy-derived datasets has enabled accurate classification of biofilm developmental stages, achieving performance comparable to expert interpretation while minimizing observer-related variability (Van Dun et al., 2025). Similarly, principal component analysis (PCA)-based methods have differentiated polymicrobial biofilms containing pathogens such as *S. aureus, P. aeruginosa*, and *A. baumannii* using transmission electron microscopy datasets (Mishra et al., 2024). Spectroscopic techniques, including Fourier-transform infrared spectroscopy combined with computational interpretation, have further enabled molecular-level characterization of extracellular matrix composition in chronic wound-associated biofilms (Mishra et al., 2024).

In addition to structural characterization, computational modeling frameworks offer mechanistic insights into biofilm development and architectural evolution. Cellular automata and individual-based models integrate microbial growth kinetics, nutrient diffusion, quorum sensing activity, and mechanical interactions to simulate density gradients, porosity changes, and structural stratification within developing biofilms (Skoneczny, 2017; Mandal et al., 2026). Experimental validation of these simulations has been achieved using time-resolved imaging techniques such as white-light interferometry, which captures biofilm growth dynamics with nanometer-scale precision (Bravo et al., 2023). More recently, genome-scale metabolic reconstructions combined with spatial simulations have enabled investigation of nutrient-driven ecological interactions and metabolic exchange within polymicrobial biofilm communities (Phalak et al., 2016).

Despite significant advances, several challenges continue to limit clinical and translational implementation. Many computational phenotyping systems rely on heterogeneous imaging protocols, limited external validation, and datasets primarily derived from simplified laboratory biofilms that may not accurately represent complex host-associated environments. Deep learning models often require large annotated datasets and substantial computational resources, while more interpretable approaches, such as PCA, may be insufficient to capture highly non-linear biological relationships. To address data scarcity, generative AI approaches, including variational autoencoders, generative adversarial networks (GANs), diffusion models, and CycleGAN frameworks, have been explored for generating synthetic annotated biofilm datasets to support downstream model training (Holicheva et al., 2025). However, synthetic data may introduce biases or artifacts that affect model performance and generalizability. Future progress will depend on standardized analytical pipelines, interoperable computational workflows, clinically representative validation datasets, and multimodal computational frameworks that integrate structural, molecular, and ecological information. Table 4 summarizes the major computational metrics and modeling approaches currently used to quantitatively characterize biofilm architecture and dynamics, along with their biological interpretation, validation context, translational maturity, and methodological limitations.

**TABLE 4:** Computational metrics and modeling approaches for quantitative analysis of biofilm architecture and dynamics.

Metric	Thickness	Biomass	Spatial heterogeneity	Matrix density	Time-lapse dynamics	Growth prediction
Methodology	Acoustic microscopy, CLSM, RGB image analysis	CLSM, gravimetric quantification, RGB analysis	CLSM, OCT	Cellular automata, individual-based computational models	White light interferometry, cellular automata simulations	Genome-scale metabolic modeling, spatiotemporal simulations
Biological interpretation	Reflects vertical structural development, maturation, and EPS accumulation	Represents total microbial burden and extracellular matrix accumulation	Captures structural irregularity, nutrient gradients, and microenvironment formation	Models EPS organization and diffusion barrier formation	Evaluates temporal growth kinetics and adaptive architectural evolution	Predicts nutrient-driven ecological interactions and spatial partitioning
Functional relevance	Influences antimicrobial penetration, oxygen diffusion, and treatment difficulty	Correlates with persistence, virulence potential, and chronic infection severity	Associated with metabolic stratification, localized tolerance, and resistance emergence	Predicts antimicrobial diffusion dynamics and resistance-associated microenvironments	Enables monitoring of treatment response and dynamic biofilm adaptation	Supports forecasting of biofilm expansion, persistence, and therapeutic response
Validation context	Primarily validated in controlled *in vitro* biofilm systems	Widely validated experimentally across laboratory biofilm models	Experimental and preclinical imaging systems	Primarily computational and simulation-based validation	Experimentally validated in time-resolved *in vitro* systems	Primarily computational and experimental validation
Organism context	Mostly mono-species bacterial biofilms	Mono- and limited mixed-species biofilms	Structured mature biofilms including polymicrobial communities	Simulated mono- and polymicrobial biofilm systems	Surface-associated laboratory biofilms	Simulated polymicrobial communities and metabolic networks
Interpretability	High	High	Moderate-High	Moderate	Moderate	Moderate
Translational maturity	Moderate translational applicability	Moderate	Emerging translational relevance	Early-stage/proof-of-concept	Emerging translational potential	Conceptual to early translational
Key limitation	Limited representation of host-associated biofilm complexity	Biomass measurements may not reflect metabolic heterogeneity or viability states	Requires advanced imaging infrastructure and standardized segmentation workflows	Simplified assumptions may inadequately capture biological and host-associated complexity	Limited integration with clinically relevant environmental and immune-associated variables.	Limited prospective clinical validation and high dependence on multi-omics data quality
References	Good et al., 2006; Lee et al., 2021; Neu and Kuhlicke, 2026	Asgharnejad et al., 2021; Lee et al., 2022	Beyenal et al., 2004; Cattò and Cappitelli, 2019	Skoneczny, 2017; Crossley et al., 2024	Skoneczny, 2017; Bravo et al., 2023	Henson, 2015; Phalak et al., 2016

## 5 Predictive modeling of antimicrobial response in biofilm systems

Accurate prediction of antimicrobial efficacy against biofilm-related infections requires analytical frameworks that integrate structural, molecular, and phenotypic determinants of tolerance. Conventional planktonic susceptibility assays often overlook adaptive traits of biofilm-associated microbes, because biofilm responses are shaped by spatial heterogeneity, metabolic stratification, and diffusion barriers. Consequently, recent predictive models incorporate multi-omics data and experimental phenotypic measurements to enhance understanding of antimicrobial activity within biofilm environments (Wannigama et al., 2020; Rajput et al., 2022; Galvez-Llompart et al., 2024).

Multi-omics approaches integrating genomics, transcriptomics, proteomics, and metabolomics have advanced understanding of resistance-associated pathways and adaptive regulatory networks that underlie biofilm persistence (Mohan et al., 2025). These methodologies facilitate the identification of genes, proteins, signaling pathways, and metabolic states associated with antimicrobial tolerance, quorum-sensing regulation, extracellular matrix production, and stress adaptation (Seneviratne et al., 2020). When combined with structural measurements and viability assays, multi-omics datasets yield a more comprehensive and biologically relevant representation of antimicrobial responses than conventional susceptibility testing alone (Kim and Rhee, 2018). Notably, these datasets elucidate how localized nutrient limitation, oxygen depletion, and metabolic dormancy contribute to heterogeneous antimicrobial susceptibility within mature biofilms.

**FIGURE 2:**
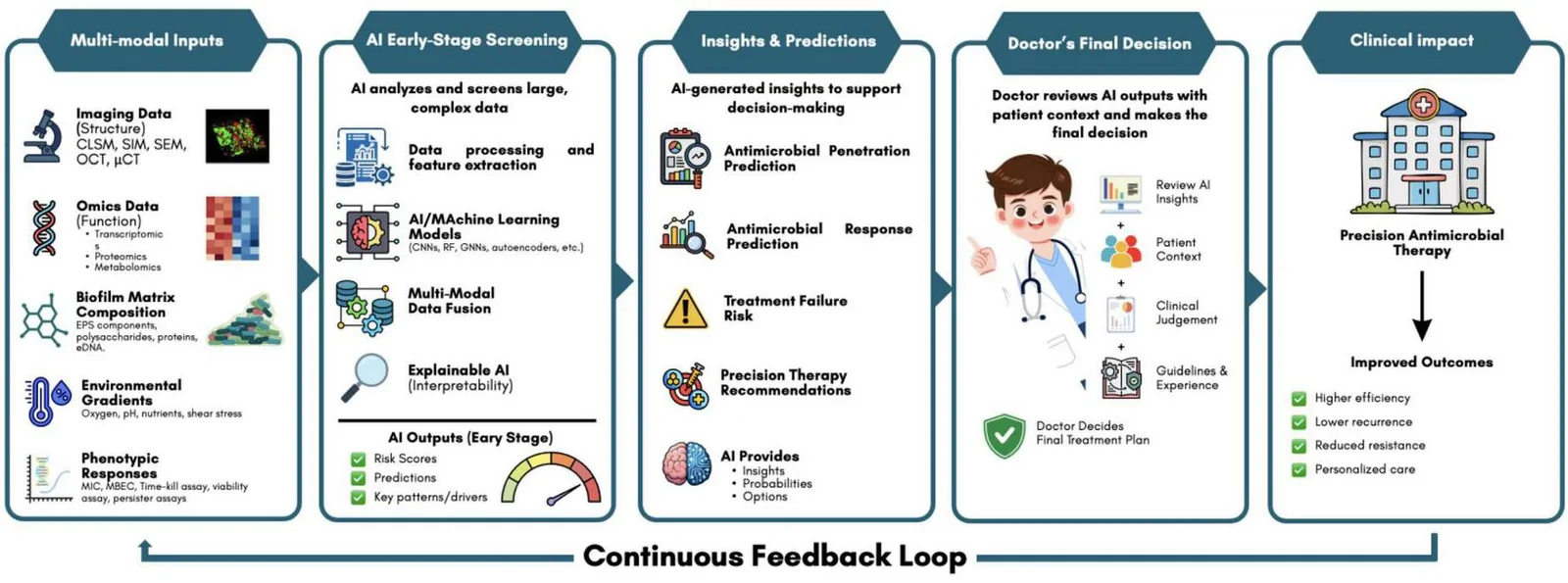
An integrated multi-modal AI pipeline for biofilm prediction and precision antimicrobial therapy.

Machine learning and deep learning systems are increasingly utilized to model complex interactions among microbial physiology, environmental conditions, and treatment outcomes. Predictive frameworks such as support vector machines, random forests, artificial neural networks, and deep learning architectures have demonstrated the capacity to analyze non-linear relationships among imaging-derived features, resistance determinants, metabolic activity, and antimicrobial exposure profiles (Grassi and Crabbé, 2024; Huang et al., 2025). These computational approaches facilitate the prediction of antimicrobial efficacy, the identification of resistance-associated phenotypes, and the prioritization of candidate anti-biofilm interventions. Complementary methodologies, including quantitative structure-activity relationship (QSAR) modeling and response surface analyses, have been applied to assess how physicochemical properties of antimicrobial compounds affect biofilm penetration and activity (Lanka et al., 2025). Analytical frameworks based on the minimal biofilm eradication concentration (MBEC) further enhance predictive assessment by incorporating biofilm-specific susceptibility measurements that more accurately reflect mature biofilm physiology than planktonic MIC testing (Busi et al., 2025). Explainable artificial intelligence (XAI) methodologies have emerged as valuable tools for improving interpretability by identifying variables most strongly associated with the emergence, persistence, and treatment failure of resistance (Rubrice et al., 2025; Tawhari et al., 2025). As shown in Figure 2, integrated multi-modal AI pipelines now combine imaging-derived structural features, omics-based molecular signatures, phenotypic susceptibility profiles, and computational modeling within unified analytical frameworks that link biofilm architecture to antimicrobial response dynamics. The major computational frameworks currently used to predict antimicrobial response in biofilm systems, together with their primary data inputs, predictive outputs, and translational considerations, are summarized in Table 5.

**TABLE 5:** Representative AI-assisted and computationally designed AMPs with potential relevance for biofilm-targeted therapeutic development.

Peptide name/type	Target pathogens	Reported activity	AI platform/design strategy	Remarks	References
Z2	*P. aeruginosa* and other bacterial pathogens	Inhibits biofilm formation and disrupts established biofilms	*de novo* computational design	Demonstrated anti-inflammatory activity and improved survival in murine pneumonia models	Zhang et al., 2025; Wu-Mo et al., 2026
HRZN-15	*A. baumannii*	Prevents biofilm formation and eradicates mature biofilms	Computational AMP design	Active against MDR isolates	Alsaab et al., 2023; Wu-Mo et al., 2026
AMP_S13	ESKAPE pathogens	Broad-spectrum biofilm inhibition	AI-designed AMP	Demonstrated *in vivo* efficacy and low toxicity	Li S. et al., 2025; Wu-Mo et al., 2026
Pep-19-mod	MDR Gram-negative and Gram-positive pathogens	Active against resistant biofilm-forming bacteria	AI-optimized AMP using *in silico* evolution	>95% bacterial reduction in murine models	Wang Y. et al., 2025; Wu-Mo et al., 2026
Mammuthusin	Gram-positive and Gram-negative bacteria	Potent antimicrobial activity	APEX deep learning platform mining extinct proteomes	Preclinical efficacy comparable to polymyxin B	Wan et al., 2024; Brizuela et al., 2025
Mylodonin	Gram-positive and Gram-negative bacteria	Broad-spectrum antimicrobial activity	APEX deep learning platform	Discovered through molecular de-extinction workflow	Wan et al., 2024; Brizuela et al., 2025
Elephasin	Gram-positive and Gram-negative bacteria	Antimicrobial activity in preclinical studies	APEX deep learning platform	Derived from extinct organism sequence mining	Wan et al., 2024; Brizuela et al., 2025
RNN-ProtTrans derived peptides	*C. acnes*	MIC 2–4 μg/mL against target pathogen	RNN generative model with ProtTrans embeddings	42 novel peptides generated, several experimentally validated	Dong et al., 2024; Brizuela et al., 2025
ProT-Diff derived peptides	Gram-positive and Gram-negative bacteria	34 of 35 synthesized peptides exhibited antibacterial activity	Diffusion model integrated with ProtT5	Demonstrated *in vivo* activity against drug-resistant *E. coli*	Wang et al., 2024; Brizuela et al., 2025

Computational diffusion modeling constitutes a critical aspect of antimicrobial prediction, as extracellular polymeric substances significantly restrict antimicrobial transport within mature biofilm systems. To address this, agent-based and continuum simulations have been developed to model antimicrobial diffusion and interaction dynamics within structurally organized biofilm matrices (Skogman et al., 2016; Bin et al., 2025; Mishra et al., 2025; Rathod and Mishra, 2026). These simulations provide computational frameworks for evaluating antimicrobial exposure across distinct biofilm regions and for predicting concentration gradients, penetration efficiency, and localized therapeutic failure within heterogeneous biofilm architectures. Furthermore, advanced delivery systems such as hydrogels and DNA tetrahedron-based nanocarriers have demonstrated improved penetration efficiency and enhanced eradication outcomes in experimental biofilm systems (Hu et al., 2025; Andresen et al., 2026). Collectively, these approaches foster a mechanistically informed understanding of biofilm-associated treatment response, moving beyond reliance on empirical antimicrobial selection alone.

Despite these advances, predictive antimicrobial modeling in biofilm systems remains limited by several methodological and translational challenges. Many existing models rely predominantly on simplified laboratory biofilms, which do not adequately capture the ecological and physiological complexity of host-associated and polymicrobial infections. Dataset heterogeneity, variability in imaging and omics workflows, and limited external validation further diminish reproducibility and generalizability across experimental settings (Hunt et al., 2005; Eick, 2021; Alcàcer-Almansa et al., 2023; Fuglsang-Madsen et al., 2025; Lanka et al., 2025; Eid et al., 2026). Additionally, clinically relevant host-associated factors such as immune interactions, fibrin deposition, tissue-specific microenvironments, and antimicrobial pharmacokinetics are often absent from training datasets, thereby restricting translational applicability. While AI-assisted predictive systems show significant promise for enhancing biofilm-oriented antimicrobial decision-making, broader implementation will require standardized validation frameworks, clinically representative datasets, prospective studies, and improved interpretability to facilitate integration into routine clinical workflows.

## 6 AI in anti-biofilm therapeutics

Computational approaches are increasingly utilized for the discovery, optimization, and translational implementation of anti-biofilm therapeutic strategies. A prominent example is the identification of halicin via deep learning-assisted molecular screening, in which computational analysis facilitated the discovery of a structurally distinct antibacterial compound with demonstrated activity against *A. baumannii* biofilms in murine wound infection models (Stokes et al., 2020). This study underscores the potential of AI-assisted screening platforms to accelerate the identification of candidate anti-biofilm compounds beyond conventional empiric discovery pipelines.

Machine learning-guided optimization of AMPs has further advanced the field of computational anti-biofilm therapeutics. These methods enable systematic exploration of peptide sequence space to identify molecules with enhanced anti-biofilm activity, reduced cytotoxicity, and improved structural stability (Wan et al., 2024). For instance, CIT-8, a computationally optimized peptide derived from citropin 1.1, demonstrated activity against methicillin-resistant and vancomycin-resistant *S. aureus* biofilms (Mishra et al., 2025). Instead of relying solely on trial-and-error experimental screening, AI-guided optimization can prioritize peptide candidates with favorable antimicrobial and physicochemical properties for subsequent validation.

Large-scale computational peptide discovery initiatives have significantly expanded the potential therapeutic landscape. For example, Santos-Júnior et al. utilized machine learning-assisted microbiome mining to develop the AMPSphere catalog, which contains over 863,000 candidate AMPs, several of which exhibited activity against biofilm-forming pathogens (Santos-Júnior et al., 2024). Generative AI systems, including protein large language models such as ProteoGPT, have also enabled *de novo* peptide design targeting biofilm-associated resistance and virulence pathways, with the goal of minimizing the development of resistance in carbapenem-resistant pathogens (Wang Y. et al., 2025). These generative frameworks demonstrate how AI can facilitate rapid hypothesis generation and molecular design in anti-biofilm drug discovery. Representative examples of AI-assisted and computationally designed AMPs with activity against clinically relevant biofilm-forming pathogens are summarized in Table 5.

Computational systems are increasingly integrated into antimicrobial decision-support platforms. Machine learning-assisted clinical decision support systems have shown potential to improve antimicrobial selection and resistance prediction compared to empirical prescribing strategies, although translational maturity and clinical validation vary across platforms (AlGain et al., 2025). More targeted, precision-oriented approaches have emerged through computational design of AMPs that selectively suppress keystone pathogens within polymicrobial biofilms. For instance, interpretable machine learning systems have been employed to develop peptides targeting *Aggregatibacter actinomycetemcomitans* in peri-implantitis-associated biofilms, with the aim of minimizing disruption of commensal microbial communities (Boone et al., 2024). These approaches indicate growing interest in microbiome-conscious and precision-oriented biofilm interventions.

Preventive applications are also gaining importance in the context of device-associated biofilm infections involving catheters, prosthetic implants, orthopedic devices, and endotracheal tubes. Factors such as surface roughness, hydrophobicity, material chemistry, and extracellular protein adsorption significantly influence microbial adhesion and subsequent biofilm maturation on biomaterial surfaces. As a result, computational modeling is increasingly used to predict bacteria–surface interactions and to optimize anti-biofilm biomaterials through surface-engineering strategies. These strategies include developing antimicrobial coatings, nanopatterned materials, and modified biomaterial surfaces to reduce microbial colonization before mature biofilms form. Computational simulations can also help evaluate how surface modifications affect microbial attachment dynamics, diffusion properties, and long-term material performance under biofilm-associated conditions.

AI-guided translational strategies have also extended to nanotechnology-assisted delivery systems and combination therapeutics. Computational optimization of nanoparticle-based carriers, hydrogels, and responsive biomaterials has shown potential to enhance antimicrobial penetration and localized drug delivery within structurally complex biofilm matrices. Concurrently, predictive modeling approaches are increasingly used to evaluate synergistic combinations of antibiotics, AMPs, bacteriophages, quorum-sensing inhibitors, and enzymatic biofilm-disrupting agents. These integrated strategies may address limitations of monotherapy and support more targeted and adaptive anti-biofilm interventions.

Despite significant conceptual promise, most computational intervention systems remain in early translational stages, with limited prospective validation and insufficient clinically representative datasets. Variability in experimental biofilm models, inconsistent benchmarking standards, limited mechanistic interpretability, and ongoing concerns about reproducibility and regulatory oversight continue to hinder clinical implementation (Li et al., 2024; Habib et al., 2025; Wang B. et al., 2025). Furthermore, many predictive systems are trained primarily on simplified laboratory conditions that do not adequately represent polymicrobial biofilms, host immune interactions, or tissue-associated microenvironments encountered in clinical infections. Therefore, rigorous validation pipelines, interdisciplinary collaboration, standardized translational assessment frameworks, and prospective clinical studies are likely required before computationally guided interventions can be widely integrated into routine biofilm management and precision antimicrobial therapy.

## 7 Translational barriers

Although AI has advanced rapidly in biofilm analysis, its adoption in clinical practice is limited by interconnected data, methodological, and implementation challenges. A key obstacle is the absence of standardized, high-quality datasets, given the substantial heterogeneity in biofilm structure, species composition, and metabolic activity. Differences in imaging modalities, experimental protocols, annotation standards, and reporting practices hinder data harmonization and compromise the robustness and generalizability of AI models (Dhaarani and Reddy, 2025; Gajendran et al., 2025). This issue is especially pronounced in antimicrobial response prediction, where clinically relevant models must account for polymicrobial interactions, spatial heterogeneity, and evolving resistance mechanisms. Consequently, many AI systems perform well in controlled settings but do not generalize effectively to diverse clinical environments, highlighting the urgent need for standardized, multi-institutional datasets with consistent annotation and validation protocols (Santhiya et al., 2025).

Another significant barrier is the limited interpretability of high-performing deep learning models, which often operate as “black-box” systems. Models such as convolutional neural networks, graph neural networks, and transformer-based architectures can achieve high predictive accuracy, yet their internal decision-making processes are difficult to elucidate (Dagnaw et al., 2025; Lavecchia, 2025). In clinical microbiology, this lack of transparency impedes adoption, as clinicians require mechanistic explanations for therapeutic decisions rather than relying solely on statistical predictions (Giacobbe et al., 2024; Cesaro et al., 2025). Although *post hoc* explainability tools such as SHAP, LIME, and Grad-CAM have been developed, their inconsistent reliability raises concerns about the trustworthiness of model interpretations (Singh et al., 2025). Moreover, interpretability in biofilm systems must encompass biological plausibility, ensuring that model outputs align with established mechanisms, including EPS dynamics, quorum-sensing regulation, and genotype-phenotype relationships. Hybrid approaches that combine rule-based reasoning with data-driven learning offer a promising avenue for enhancing both interpretability and predictive reliability (Cavallaro et al., 2023; Boone et al., 2024; Sufi, 2025).

In addition to technical challenges, the absence of prospective clinical validation substantially hinders the implementation of AI-driven biofilm models in clinical practice. Most existing studies use retrospective datasets or controlled experimental systems that do not adequately capture the complexity of real-world biofilm-associated infections, including host-pathogen interactions and variability in treatment responses (Dong et al., 2025). As a result, the clinical utility of AI-based predictions, especially for antimicrobial therapy guidance, remains insufficiently established. This limitation is further exacerbated by regulatory and ethical issues, including data privacy, algorithmic bias, and a lack of standardized evaluation protocols (Non et al., 2025). Regulatory bodies are increasingly prioritizing transparency, accountability, and ongoing monitoring. For example, recent FDA guidance on AI-enabled medical software specifies requirements for data provenance, performance monitoring, and bias mitigation, while the European Union AI Act designates medical AI systems as high-risk applications that require stringent governance and human oversight (European Union, 2024; U.S. Food and Drug Administartion, 2024; U.S. Food and Drug Administartion, 2026). These trends indicate that successful clinical integration of AI will require not only strong predictive performance but also trustworthiness, reproducibility, and adherence to regulatory standards (Sande and Rajguru, 2025).

**FIGURE 3:**
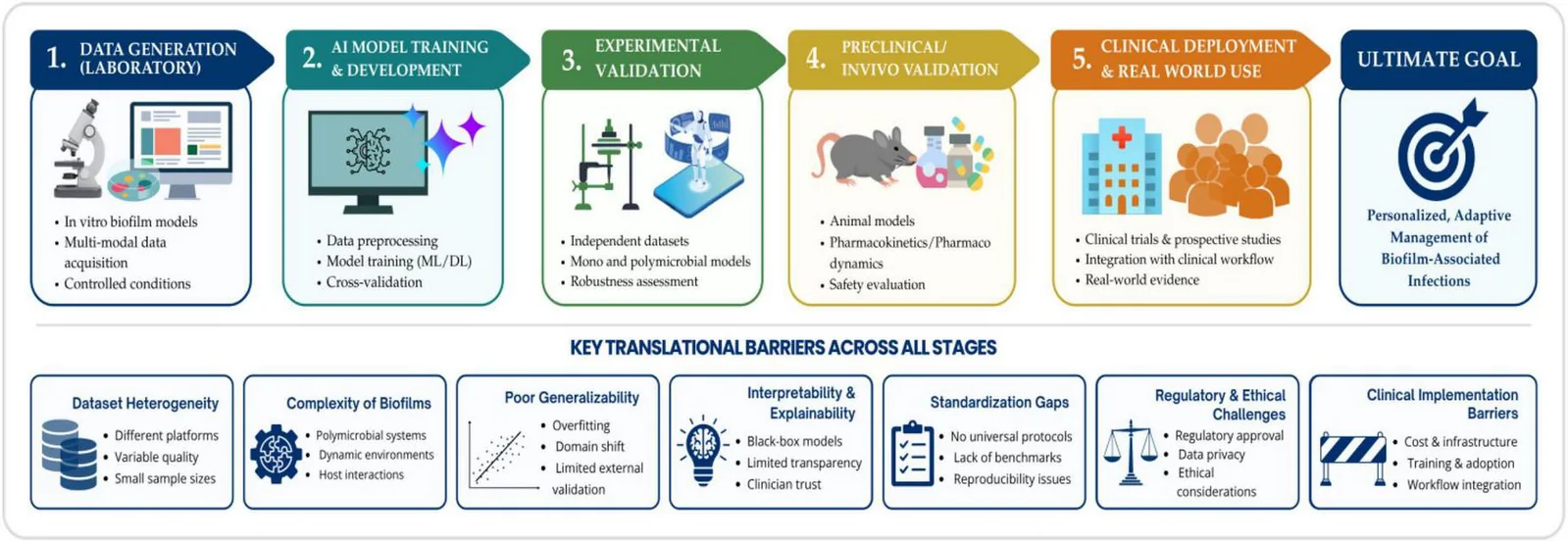
Translational roadmap and clinical barriers for AI-guided biofilm management.

These translational barriers also illustrate a broader systems-level fragmentation in current AI applications for biofilm research, in which detection, structural quantification, and antimicrobial prediction are often treated as separate analytical tasks rather than as components of integrated computational frameworks (Wu et al., 2025). This fragmentation impedes the development of clinically relevant decision-support systems that can link biofilm characterization to therapeutic optimization. Overcoming this issue will require the creation of end-to-end, multi-modal pipelines that integrate imaging, omics, phenotypic, and clinical data within unified predictive architectures. Future research should focus on generating standardized datasets, developing biologically interpretable AI, and conducting prospective multi-center validation studies to assess performance across diverse clinical biofilm environments (Hao et al., 2025). Additionally, digital twin concepts that integrate patient-specific biological data with computational simulations may provide a future framework for predictive, precision-based antimicrobial management. Figure 3 summarizes the major translational stages, implementation barriers, and future directions for AI-guided biofilm management.

## 8 Future prospect

Many current computational frameworks rely on heterogeneous datasets, lack rigorous external validation, and employ laboratory biofilm models that do not adequately capture the complexity of polymicrobial and host-associated biofilm communities (Mishra et al., 2024; Xu et al., 2025; Bhattacharya et al., 2026). Advancing this field necessitates the development of standardized imaging protocols, interoperable biofilm databases, and benchmark datasets that integrate structural, molecular, metabolomic, and phenotypic information. The integration of machine learning and deep learning with confocal microscopy, optical coherence tomography, and multi-omics technologies can improve the characterization of biofilm architecture, metabolic heterogeneity, and antimicrobial susceptibility patterns (Vaz and Balaji, 2021; Habib et al., 2025; Verma et al., 2025; Wang Y. et al., 2025; Bhattacharya et al., 2026; Dutta and Ray, 2026; Talianu et al., 2026). Additionally, the adoption of XAI frameworks may enhance model interpretability and increase confidence in AI-assisted analytical workflows (Xu et al., 2025; Talianu et al., 2026). AI offers significant potential to advance the discovery and optimization of antibiofilm interventions. Recent research has investigated its use in antimicrobial peptide design, drug repurposing, and predictive modeling of antimicrobial responses, though most methods remain at the preclinical stage (Abbara et al., 2025; Tyagi et al., 2025; Bhattacharya et al., 2026; Talianu et al., 2026). Advances in generative AI, protein language models, and computational peptide engineering are likely to accelerate the identification of novel therapeutic candidates targeting biofilm-associated pathogens. Additionally, integrating biofilm imaging, antimicrobial resistance profiles, host-response biomarkers, and ecological interaction data may enable more personalized treatment strategies. Progress in computational simulation and digital twin technologies could further support investigations of biofilm development and treatment responses under dynamic conditions (Phalak et al., 2016; Skoneczny, 2017; Bravo et al., 2023; Mandal et al., 2026). Nevertheless, extensive experimental validation, including studies with clinically relevant biofilm models and translational research, is required to establish the practical utility of these approaches. Overall, these advancements indicate that AI is poised to become an increasingly important element in biofilm research and therapeutic development.

## 9 Conclusion

Biofilm-associated infections are challenging to manage because conventional diagnostic and antimicrobial susceptibility frameworks do not fully capture their structural heterogeneity, metabolic variability, or adaptive ecological behavior. Recently, AI and computational modeling have demonstrated increasing potential to integrate imaging, multi-omics, phenotypic, and computational datasets to detect biofilms, characterize structures, and analyze antimicrobial responses. Advances in machine learning, deep learning, explainable AI, and computational modeling have improved understanding of how biofilm architecture and microbial physiology influence antimicrobial tolerance and treatment outcomes. However, significant translational and methodological limitations persist. Many current AI systems rely on simplified *in vitro* datasets that do not adequately represent polymicrobial organization, host-associated interactions, or the ecological complexity of clinical biofilms. Consequently, strong computational performance does not necessarily ensure biological generalizability or clinical applicability. Furthermore, several predictive frameworks remain primarily correlation-based, which limits mechanistic interpretability and broader translational relevance. Future progress will depend on the development of standardized datasets, biologically informed and interpretable models, prospective validation studies, and stronger integration between computational sciences and mechanistic biofilm research.
